# On the Heat Stability of Amyloid-Based Biological Activity: Insights from Thermal Degradation of Insulin Fibrils

**DOI:** 10.1371/journal.pone.0086320

**Published:** 2014-01-21

**Authors:** Weronika Surmacz-Chwedoruk, Iwona Malka, Łukasz Bożycki, Hanna Nieznańska, Wojciech Dzwolak

**Affiliations:** 1 Institute of Biotechnology and Antibiotics, Warsaw, Poland; 2 Institute of High Pressure Physics, Polish Academy of Sciences, Warsaw, Poland; 3 Nencki Institute of Experimental Biology, Polish Academy of Sciences, Warsaw, Poland; 4 Department of Chemistry, University of Warsaw, Warsaw, Poland; University of Maryland School of Medicine, United States of America

## Abstract

Formation of amyloid fibrils in vivo has been linked to disorders such as Alzheimer’s disease and prion-associated transmissible spongiform encephalopathies. One of the characteristic features of amyloid fibrils is the high thermodynamic stability relative both to native and disordered states which is also thought to underlie the perplexingly remarkable heat resistance of prion infectivity. Here, we are comparing high-temperature degradation of native and fibrillar forms of human insulin. Decomposition of insulin amyloid has been studied under helium atmosphere and in the temperature range from ambient conditions to 750°C using thermogravimetry and differential scanning calorimetry coupled to mass spectrometry. While converting native insulin into amyloid does upshift onset of thermal decomposition by ca. 75°C, fibrils remain vulnerable to covalent degradation at temperatures below 300°C, as reflected by mass spectra of gases released upon heating of amyloid samples, as well as morphology and infrared spectra of fibrils subjected to incubation at 250°C. Mass spectra profiles of released gases indicate that degradation of fibrils is much more cooperative than degradation of native insulin. The data show no evidence of water of crystallization trapped within insulin fibrils. We have also compared untreated and heated amyloid samples in terms of capacity to seed daughter fibrils. Kinetic traces of seed-induced insulin fibrillation have shown that the seeding potency of amyloid samples decreases significantly already after exposure to 200°C, even though corresponding electron micrographs indicated persisting fibrillar morphology. Our results suggest that amyloid-based biological activity may not survive extremely high temperature treatments, at least in the absence of other stabilizing factors.

## Introduction

Association of misfolded globular proteins may lead to the emergence of highly ordered β-sheet-rich linear aggregates, so-called amyloid fibrils. Presence of amyloid deposits in human tissues has long been linked to several degenerative disorders such as Alzheimer’s, Parkinson’s, Huntington’s diseases and the prion-associated Transmissible Spongiform Encephalopathies (TSEs) including Creutzfeldt-Jakob disease [1]–[3]. On the other hand, there are known examples of nonpathogenic and even biologically-functional amyloid fibrils [4]–[5]. Because amyloid-like structures may be induced in vitro in many unrelated globular proteins [6], polymerized α-amino acids [7], and even short synthetic dipeptides [8] it was proposed that formation of these aggregates reflects a common generic feature of proteins as polymers [7]. Amyloid fibrils from different precursors share a high degree of similarity in terms of the high β-sheet content (regardless of precursor’s native conformation), morphology, tinctorial properties and autocatalytic character of their self-assembly [9]–[10]. The latter observation prompted the idea that the amyloid state may correspond to protein’s global minimum of free energy [11]. This concept is supported by the fact that de novo nucleation of amyloid usually requires conditions disrupting the native state while the subsequent stage of elongation of fibrils typically continues even under non-denaturating conditions. The thermodynamic stability of fibrils is paralleled by their marked resistance to high temperatures [12], hydrostatic pressure [13], denaturants [14] and proteolytic enzymes [15]. Likewise, an unusual stability against denaturating factors including high temperatures and proteolytic enzymes hallmarks prion infectivity [16]–[19]. As a consequence, prions are resistant to standard clinical decontamination protocols and may survive years in environment (e.g. in soil) posing enormous challenges in medicine and agriculture. In response to this problem, novel effective ways of eradication of prions – for example through the application of extremely high pressures [20]–[22] – are urgently sought after. Still, the reported degree of high temperature resistance of prions is puzzling. While autoclaving, i.e. moderate heat treatment at ca. 134°C in the presence of water, is generally effective for prion inactivation [23], and parallels denaturation of amyloid fibrils from nonpathogenic proteins taking place under similar conditions (e.g. [12]), prions in the dried state have been reported to withstand much higher temperatures. According to Brown et al., prion infectivity partly survives ashing at 360°C [24]. In following study, researchers from the same group claimed that hamster-adapted TSE agent would remain partly infectious even after ashing at 600°C [25]. As no biological matrix is expected to survive such an extreme thermal treatment, the authors argued that the prion amyloid undergoes high-temperature-induced mineralization and converts into inorganic heat-resistant template capable of imprinting and spreading pathogenic isoforms of PrP^C^ (normal cellular isoform of prion protein). However, such hypothetical process could pass structural features of the organic prion to its inorganic replica only on a rather “low-resolution” morphological level – i.e. even if the fibril’s shape were retained throughout, surface chemical groups of amino acid side chains would vanish. This would necessarily have dramatic consequences for the molecular recognition between the now-inorganic “prion” and incoming PrP^C^ monomers – i.e. process governing spread of prion infectivity at the atomic level. Even more likely, no shape-preserving mineralization would take place at temperatures as high as employed by Brown et al. since such conditions promote robust structure-disrupting chemical reactions within the heated protein. Here, we examine high-temperature stability of insulin amyloid fibrils in light of the contemporary paradigm linking propagation of prions and spread of amyloid fibrils from other proteins [9], [26]–[27]. Insulin has proved to be an insightful model for studies on protein aggregation [28]–[30] including certain aspects of prion-like polymorphism of amyloid fibrils [14], [31].

In this work, insulin amyloid is subjected to high temperature treatment under strictly controlled conditions afforded by TGA (Thermogravimetric Analysis) apparatus. This approach has several advantages, namely: [i] covalently homogenous samples of fibrils from recombinant protein are analyzed; [ii] heating is carried out under atmosphere of inert gas – helium – which prevents undesirable side processes such as spontaneous combustion; [iii] mass of fibril sample, heat effects, and chemical composition of gases released during heating can be probed with high accuracy. After heating, samples were subjected to usual morphological and structural analysis. In vitro seeding potency of heated insulin fibrils is used as a yardstick of surviving biological activity – understood as structural and chemical compatibility of the amyloid seed and its globular precursor on the amyloidogenic pathway.

## Materials and Methods

### Preparation of Fibrils

Human insulin was manufactured at the Institute of Biotechnology and Antibiotics (Warsaw, Poland) using recombinant DNA technology [32]. Native insulin used in this study contained 2.7 Zn^2+^ cations per 6 monomers. Insulin amyloid fibrils were obtained through a quiescent incubation of 1 wt. % insulin solution in 0.1 M NaCl in H_2_O, pH 1.9 (adjusted with diluted HCl) at 65°C for 24 h. The thus obtained fibrils were sonicated (by applying 2-second-long pulses 60 times) using a Sonics VCX 130 Ultrasonic Processor (USA) operating at 20% of its maximum power output and 20 kHz frequency. In order to precipitate fibrils efficiently and remove excess of hydrochloric acid, pH was raised closer to the isoelectric point of insulin (5.5) – by adding diluted NaOH till it reached 6.0. Subsequently, samples were centrifuged for 30 min at 13 000 rpm. After removal of supernatant, viscous gel was washed with deionized water and centrifuged again. Procedure was repeated ten times in order to remove traces of NaCl; afterwards samples were frozen in liquid nitrogen and subsequently freeze-dried for 20 h using FreeZone 1 Liter Benchtop Freeze Dry System, Labconco until gas pressure over the sample stabilized below 0.05 mbar (at 20°C).

### Thermal Analysis

TGA MS (Mass Spectrometry) and DSC (Differential Scanning Calorimetry) experiments were conducted using an STA 449 F1 Jupiter apparatus from NETZSCH-Feinmahltechnik GmbH, Germany. Approximately 6 mg portions of dry samples were placed in alumina crucible and heated to 750°C at a constant rate of 10°C/min and under continuous helium flow. Under these conditions the TGA/DSC/MS experiments on insulin samples were sufficiently reproducible, as is demostrated by repetitive scans placed in Supporting Information (Figure S1). Released gases were analyzed on 403 Aëolos quadrupole mass spectrometry (QMS) module from NETZSCH-Feinmahltechnik GmbH, Germany operating at 70 eV ionization electron energy. For qualitative analysis of released gases Multiple Ion Detection (MID) mode was used.

### High-temperature Incubation

Experiments were performed using the same equipment as for TGA/DSC analysis: approx. 6 mg portions of powdered samples were put into alumina crucible and heated to: 100, 200, 250, 290, 350 or 750°C at constant rate of 10°C/min under helium flow. After desired temperature was reached, isothermal conditions were held for 10 minutes. At the end, samples were gradually cooled down to room temperature, manually grinded, and suspended in aqueous 0.1 M NaCl, pH = 1.9 - for FT-IR (Fourier Transform Infrared) spectroscopy and seeding experiments - or in neat H_2_O for TEM/SEM (Transmission Electron Microscopy/Scanning Electron Microscopy) analysis - and sonicated afterwards, as in the case of freshly prepared fibrils.

### FT-IR Spectroscopy

About 20 µl of fibrils suspension was dropped on to CaF_2_ window, and dried at room temperature. All transmission FT-IR spectra of thus obtained film deposits were collected on Nicolet NEXUS FT-IR spectrometer equipped with a liquid nitrogen-cooled MCT detector. For a single spectrum 256 interferograms of 2 cm^−1^ resolution were co-added. During measurements the sample chamber was continuously purged with dry CO_2_-depleted air. All insulin spectra were baseline-corrected. Data processing including calculation of second derivative infrared spectra (Savitzky-Golay) was performed using GRAMS software (ThermoNicolet, USA).

### Morphology Analysis

**TEM.:** The morphology of the fibrils was examined on JEM 1400 transmission electron microscope from JEOL Co., Japan, equipped with a high resolution CCD MORADA, SiS-Olympus digital camera. Copper grids (400 mesh) covered with collodion (SPI Supplies, West Chester, PA, USA) and carbon were used. A 10-microliter sample (1 mg/ml) was applied to a grid for 40 s and then negatively stained for 25 s with 2% (w/v) uranyl acetate (SPI Supplies, West Chester, PA, USA). Grids were dried at room temperature prior to imaging.

**SEM.:** For SEM analysis, droplets of aqueous suspensions of samples were deposited on silicon wafers and dried under vacuum (approx. 0.05 mbar) at ambient conditions. Dried films were sputtered with approximately 10-nm-layer of Au/Pd alloy before SEM images were collected on Zeiss ULTRA plus microscope.

### Kinetic Measurements of Thioflavin T Fluorescence

To 1 wt. % freshly prepared solutions of human insulin in 0.1 M NaCl, pH 1.9, ThT (Thioflavin T) was added to the final concentration of the fluorophore of 20 µM. Afterwards, examined fibril samples (before or after temperature treatment) were suspended in 0.1 M NaCl, pH 1.9, sonicated (using the same routine as for freshly grown amyloid samples for TGA/DSC measurements) and added to the native insulin solution at 1∶ 100 dry seeds : native insulin ratio. Measurements were carried out using Fluoroskan Ascent FL fluorometer equipped with a pair of λ_ex._ 440 nm/λ_em._ 485 nm optical filters and 96-well black microplates. Aggregation of insulin samples was monitored by probing intensity of ThT fluorescence excited at 440 nm. Kinetic experiments were carried out at 37°C and gentle agitation at 300 rpm. In order to assess reproducibility of aggregation kinetics, 6 microplate wells were filled with 170 µl portions of each sample for parallel measurements.

## Results and Discussion

**Figure 1:**
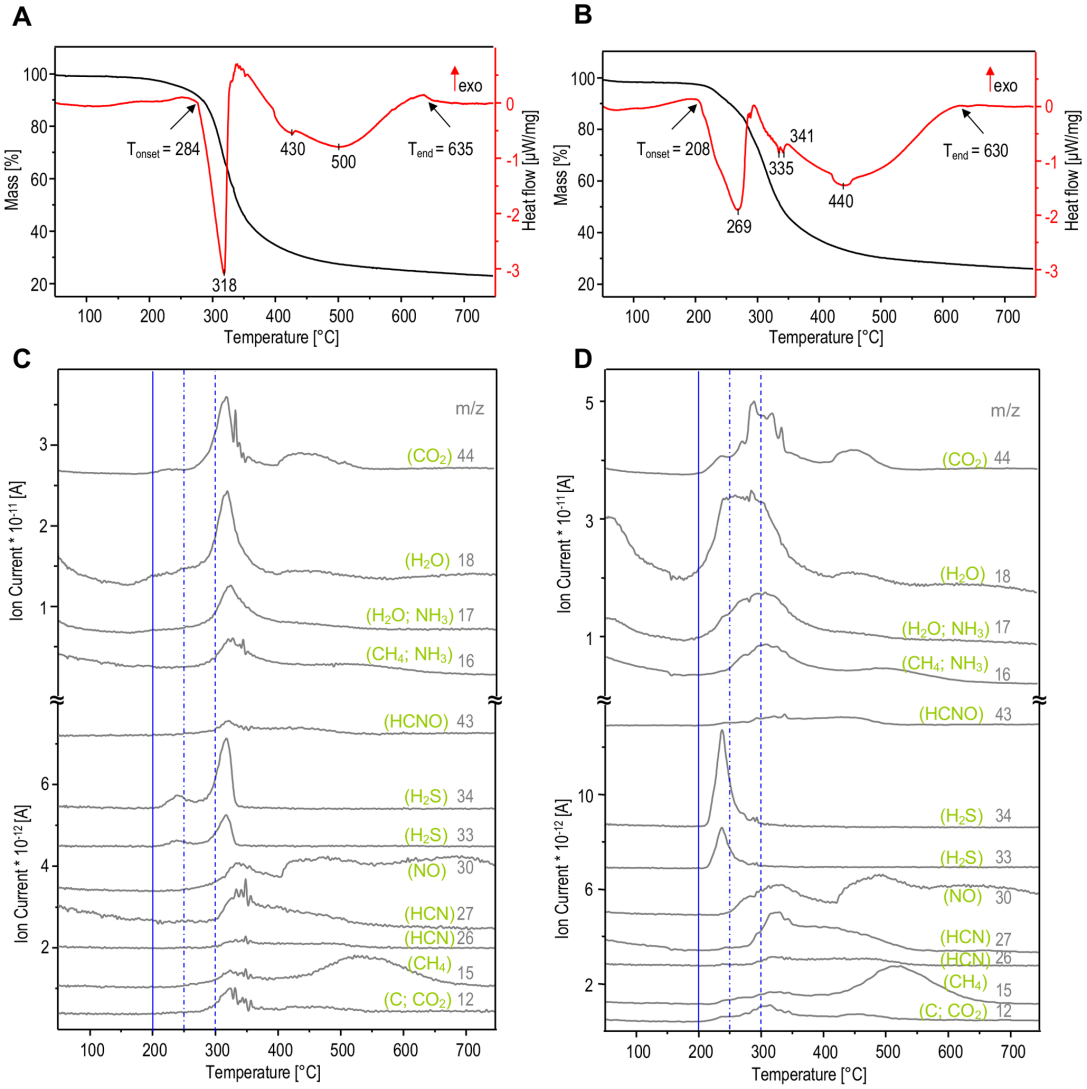
Thermal stability of human insulin in the amyloid (A, C) and native (B, D) states. Panels A and B presents thermogravimetric curves (black lines) together with the corresponding heat flow profiles (red lines) obtained through heating of samples form 50 to 750°C with a linear ramp 10°C/min under helium atmosphere. Panels C and D show TGA-MS evolution profiles of gases released during thermal analysis at selected m/z channels and assigned to different molecules.

Thermogravimetric (TGA) and differential scanning calorimetry (DSC) curves of amyloid fibrils prepared from human insulin are shown in Figure 1A. The aggregate remains stable up to approximately 284°C when a sharp endothermic transition with its peak at 318°C sets in. The simultaneous loss of ca. 60% of mass is reflected by sigmoidal shape of the TGA curve. A second much broader endothermic transition accompanied by less significant loss of mass is detected by DSC between 350°C and 635°C with its peak at ca. 500°C. Comparison to the native human insulin (Figure 1B) indicates that both forms undergo similar temperature-induced processes, as detected by TGA and DSC. However these transitions are much more cooperative and shifted to higher temperatures by roughly 75°C in the case of fibrils confirming that the amyloid conformation stabilizes insulin against heat. The dramatic loss of mass of heated amyloid coinciding with the first endothermic peak implies that the underlying transition must involve profound modification of the covalent skeleton of fibrillar insulin. Interestingly, the DSC and TGA signals are less coupled for the native protein. For heated insulin fibrils, the second high-temperature endothermic peak is more likely to stem from physicochemical transitions taking place in the solid state without a significant release of gases as is corroborated by flattening TGA curves.

Coupling of TGA/DSC apparatus to MS employed in this study has allowed us to analyze gases released upon heating of amyloid (Figure 1C) and native insulin (Figure 1D) samples. The ion currents measured at different m/z ratios and assigned to original and fragmented molecular ions reflect the changing chemical composition of the gases released upon heating. The best resolved and most intensive signals are for CO_2_ (m/z = 44), H_2_O (m/z = 18 and 17), NH_3_ (m/z = 17 and 16), and H_2_S (m/z = 34 and 33). Importantly, appearance of all these peaks is strongly cooperative and correlated with the first endothermic DSC peak at 318°C (Figure 1A). The MS signals corresponding to the second DSC-detected transition are primarily related to release of CO_2_ and CH_4_ (Figure 1C and 1D). Hence the first temperature-induced transition in insulin amyloid fibrils must involve substantial decomposition not only of protein structure as such but also of its building blocks. That the major water signal in MS evolution profiles is synchronized with the releases of other gases suggests that these H_2_O molecules are not loosely-bound water of crystallization, but rather a product of chemical decomposition of protein. Temperature-induced release of trapped water molecules has been reported for peptide nanotubes self-assembled from diphenylalanine [33]. On the other hand, presence of water-filled canals within amyloid fibrils has been postulated by Perutz et al. [34]. However, in accordance with our previous spectroscopic study [35], the current result on insulin fibrils shows no evidence of such water molecules.

**Figure 2:**
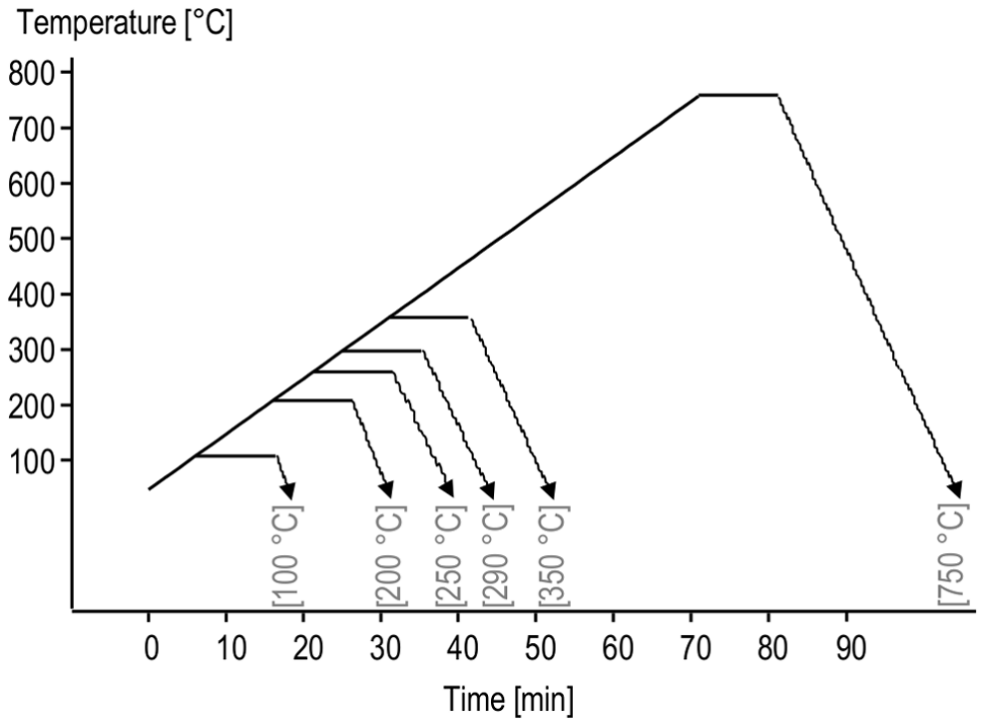
Temporal profiles of high temperature incubations of amyloid samples. Powder sample were heated to 100, 200, 250, 350°C with a linear ramp 10°C/min under helium atmosphere. After desired temperature was reached, isothermal conditions were held for 10 minutes, followed by gradual cooling of the samples down to room temperature.

**Figure 3:**
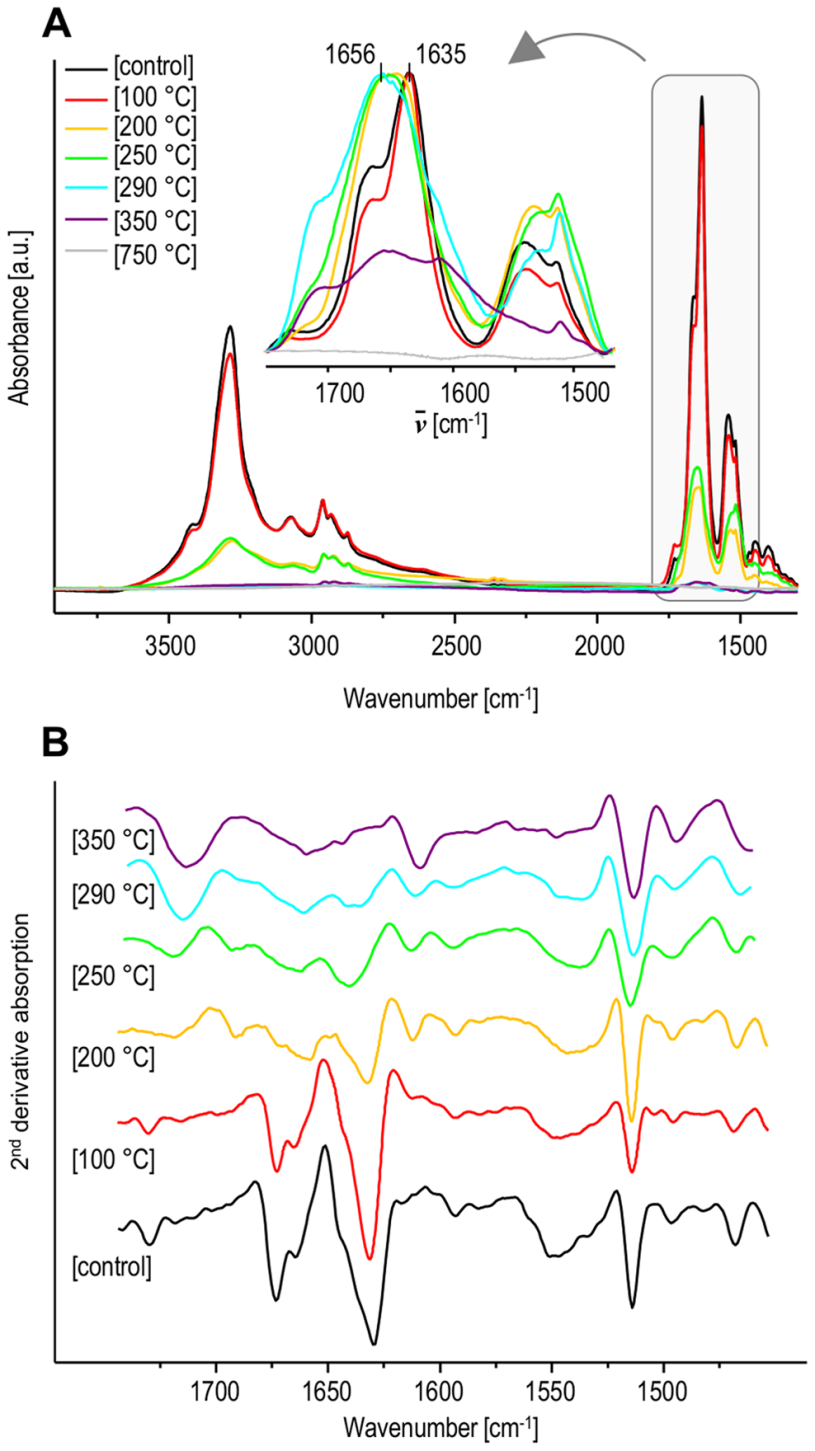
Infrared absorption (A) and second derivative (B) spectra of amyloid fibrils before and after exposure to high temperatures. Inset in panel A shows normalized spectra in the amide I/II spectral region. Second derivative FT-IR spectra in panel B are shown only for the amide I/II region with omission of featureless 750°C spectrum.

Compared to the case of insulin fibrils, the release of gaseous products of the thermal decomposition of native insulin starts at lower temperatures and is significantly less cooperative (Figures 1D versus 1C). The early increase of signals at m/z = 18 and 17 is likely to reflect release of water of crystallization apparently more abundant in native than in fibrillar insulin. Maxima of ion currents at m/z = 34 and 33 (H_2_S) occur even before the temperature reaches 250°C implying very early degradation of insulin’s disulfide bridges which are argued to link the same Cys side chains in both native and fibrillar insulin [36]. The TGA and DSC scans highlighted major temperature-induced transitions in insulin amyloid (Figure 1) which enabled selection of appropriate heating profiles for following isothermal incubations of fibrils (shown in Figure 2). The high-temperature incubations were carried out using TGA apparatus and under constant flow of helium in order to prevent oxidation-induced damage of samples. After cooling down to room temperature, samples were subjected to further spectroscopic and microscopic analysis. Figure 3 presents FT-IR transmission spectra of CaF_2_-deposited films of insulin fibrils heated at different temperatures. The overall decrease in intensity of infrared bands (most conspicuously starting with 200°C-treated samples) is caused by both decomposition of protein covalent structure and occurrence of strongly light-absorbing carbon (upon heating, samples gradually change color from white to yellow, then brownish, and black). The conformation-sensitive amide I and II vibrational bands are shown in inset of Figure 3 while corresponding second-derivative spectra (up to 350°C) are placed in panel 3B. According to the infrared data, incubation at 100°C has practically no effect on fibrils, as the corresponding amide I band remains at ca. 1635 cm^−1^– position assigned to non-deuterated parallel β-sheet conformation typical for insulin fibrils [35]. However, the exposure to 200°C already causes a profound disruption of the secondary structure with the band shifting toward 1656 cm^−1^ and broadening significantly, at the same time. Although further spectral changes occur with increasing temperature of incubation, a small band assigned to tyrosine side chains at around 1516 cm^−1^ persists up to 350°C.

**Figure 4:**
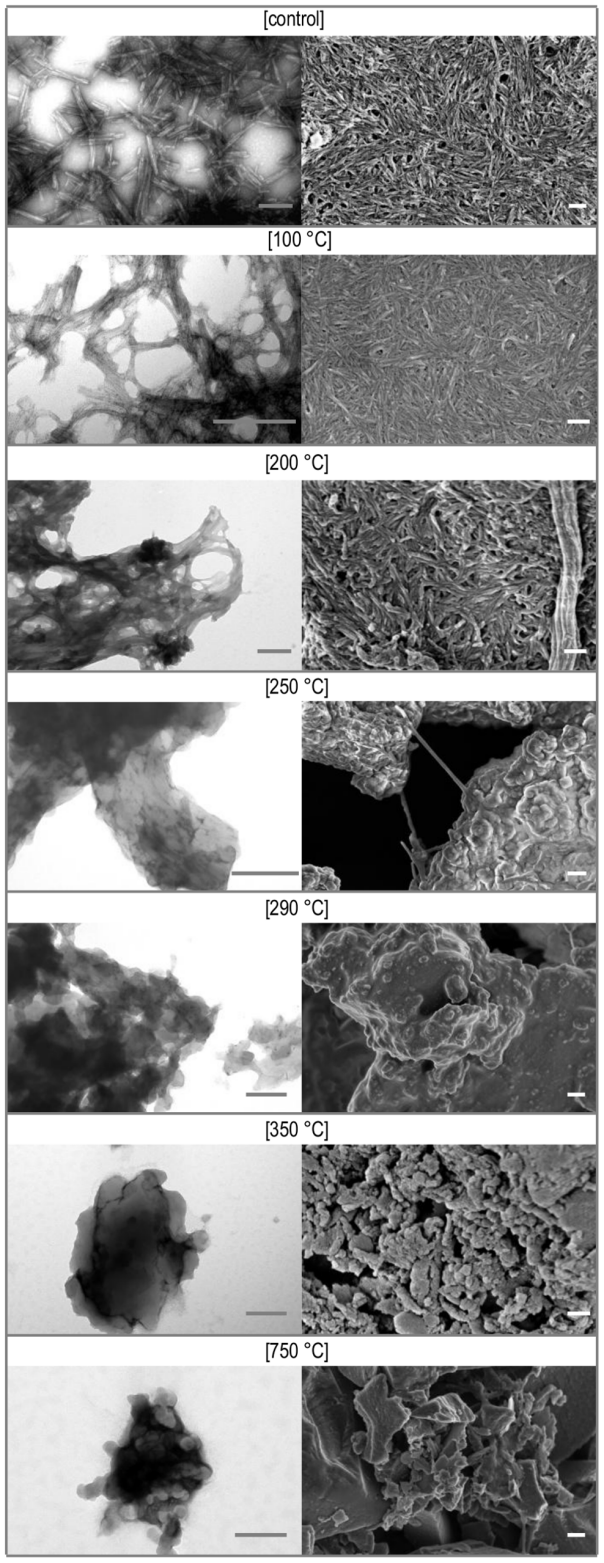
TEM (left) and SEM (right) images of amyloid fibrils before and after heating. Scale bars are 200 nm.

We have also employed TEM and SEM techniques in order to assess changes in fibrillar morphology caused by the high temperature treatment (Figure 4). SEM images (right panel) do not reveal any significant difference between untreated fibrils and those exposed to 100°C but the corresponding TEM images suggest further association of individual fibrils caused by the heat treatment. At 200°C fibrils appear swollen and melting in SEM, while the degree of association in the corresponding TEM images makes observation of single specimen no longer possible. These changes remind one of a similar course of temperature-induced changes in morphology of diphenylalanine nanotubes reported earlier [33], [37]. Further increase of temperature up to 250°C leads to the complete disarray of the fibrous scaffold: samples become entirely amorphous.

**Figure 5:**
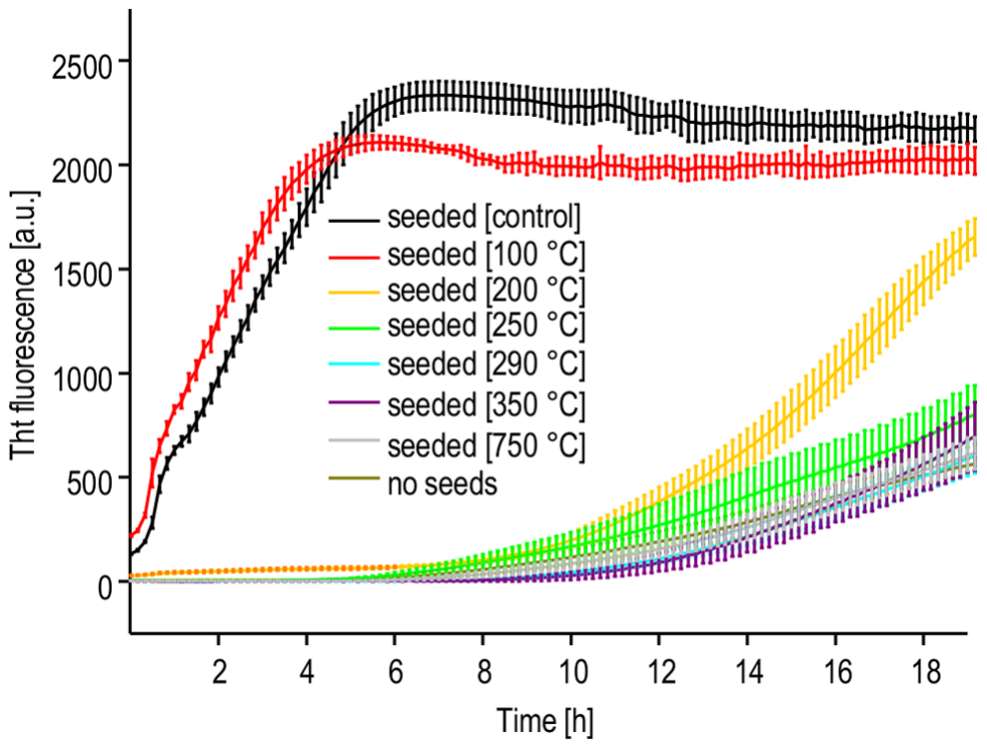
Aggregation kinetics of human insulin seeded at 37°C with fibrils exposed to high temperatures, probed by ThT fluorescence. Error bars correspond to standard deviations of fluorescence intensity calculated for six microplate wells with identical samples.

Apart from conformational and morphological traits, one of the key features of amyloid fibrils is the ability to catalyze its own proliferation at the expense of correct native form – either in vitro or in vivo. A very straightforward way to probe seeding capacity of insulin fibrils is to monitor kinetics of insulin aggregation in their presence. At moderate temperatures, spontaneous insulin aggregation is preceded by long lag-phase which is cut short even in the presence of traces of preformed insulin fibrils. Kinetics of the process can be probed conveniently by ThT fluorescence using microplate reader [14]. We have used this approach to compare seeding potency of insulin fibrils before and after exposure to the same heating regimes as used for FT-IR, TEM and SEM analysis. Under the conditions of this experiment specified in Materials and Methods section, insulin aggregation occurs after a lag time of approximately 7 hours (average from six wells of the microplate). However, in the presence of sonicated insulin fibrils (added at the 1∶100 ratio to the total insulin mass in solution) the process becomes very fast and no lag phase is observed. The kinetic traces in Figure 5 show that 100°C treatment of seeds has no decelerating effect on insulin aggregation, while the seeding strength of amyloid exposed to 200°C is significantly compromised. This tendency continues for fibrils heated to even higher temperatures. Hence, the kinetic data captures one more aspect of temperature-induced degradation of amyloid fibrils: loss of the capacity to seed daughter fibrils. It should be stressed that the heated amyloid samples are sonicated after the high-temperature incubation. In other words, degradation must be taking place in bulk protein and not only on fibrils’ surfaces, as otherwise sonication treatment – by breaking fibrils into short pieces – would release “fresh” and active ends and restore seeding potency.

Our study has highlighted several aspects of thermal decomposition of dry insulin amyloid fibrils. Insulin molecules are stabilized to a degree by being built into amyloid scaffold (compared to the native state) but this effect is rather limited as the covalent structure of fibrils undergoes degradation below 300°C. Although the morphology of insulin amyloid appear to withstand incubation at 200°C, such treatment dramatically reduces its “biological activity” – i.e. the capacity to induce daughter amyloid afterwards. This non-cooperativity is understandable in light of strict requirements of structural and chemical compatibility between surfaces of amyloid and recruited protein for the correct molecular recognition and docking events to take place and enable fibril’s elongation: a tiny disruption of the chemistry of amino acid side chains may switch off the seeding effect, even if the overall supramolecular architecture remains intact. High levels of chemical compatibility between the soluble protein and the seed are conditions sine qua non of tip-to-tip elongation, but there could be another less demanding fibrillation route – the one more likely to survive high temperature treatment: secondary nucleation pathway [28]–[29], [38]. Native insulin tends to unfold and aggregate (forming amyloid fibrils) at variety of hydrophobic surfaces. Hypothetically, this process could take place at the interface between protein solution and charred organic particles regardless of their amyloid (or not) origins. Within the accuracy of the kinetic experiments no evidence of such effects is seen (Figure 5).

Previous works have indicated that subtle covalent modifications – e.g. crosslinking – are taking place in dry proteins heated at moderate temperatures [39]–[40]. Similar processes are likely to occur in gradually heated amyloid fibrils. In the presence of other substances (for example reducing sugars), additional phenomena (e.g. Maillard reaction) may contribute to covalent modification of heated protein. Suyama et al. have depicted such scenario for thermal inactivation of prions [41]. This study has been conducted under conditions limiting additional processes that could lead to protein degradation: namely in the absence of oxygen and reducing agents – in order to capture possibly highest threshold of thermal stability of amyloid fibrils. While analyzing relevance of these results for insulin fibrils in the context of the levels of heat resistance claimed for prions [25], one must spell out certain limitations of our approach, as well as experimental differences between our work and Gajdusek’s studies. First of all, insulin amyloidogenesis is not a proper in vitro model of prion diseases, although its end product may share certain general structural and physicochemical traits with prions. Insulin fibrils have been chosen here for their accessibility and well-defined composition. On the other hand, previously published heating effects on prion effectivity were not examined using pure scrapie protein, but crude brain homogenates [24]–[25]. In principle, such homogenates could contain substances stabilizing to a degree the prion protein. In our opinion, this scenario would be more likely for protein denaturation in solution and in moderate temperature range when additional stabilization of globular proteins is often achieved through desolvation. In the dry state, and at hundreds of Celsius degrees processes triggered by reactive excited electronic states would contribute predominantly to protein decomposition. The concept of preventing high-temperature decomposition through weak interactions with third bodies (components of homogenated tissue) seems a bit speculative from the chemical point of view. Another important difference between this study and Gajdusek’s works concerns the detection method of the biological activity surviving heat treatment. Limit of detection of the biological assays determining level of prion infectivity (which cannot be used in studies on noninfectious insulin amyloid) is orders of magnitude below the detection threshold of in vitro seeding experiments carried in this work. Therefore for all these limitations, our study attempts to gather the missing reference data on heat stability of amyloid seeds in the dry state, rather than to directly challenge findings by Gajdusek et al. [24]–[25].

## Conclusions

In summary, thermal degradation of dry insulin fibrils requires temperature higher by ca. 75°C than degradation of native insulin. Covalent decomposition of amyloid begins below 300°C and, unlike for the native form, leads of highly cooperative release of water, carbon dioxide, ammonia, hydrogen sulfide, among other gases, along with concomitant dismantling of secondary structure and morphology of fibrils. Seeding potency of insulin amyloid appears to diminish even before this temperature range is reached. This study suggests that amyloid-supported biological activity of pure fibrils may be more sensitive to high temperatures than it is often assumed.

## Supporting Information

Figure S1 **Repeated TGA/DSC scans of human insulin amyloid samples.**
(PDF)
