# Supplementary material for: On the Heat Stability of Amyloid-Based Biological Activity: Insights from Thermal Degradation of Insulin Fibrils
Source: PLoS One. 2014 Jan 21;9(1):e86320. doi: 10.1371/journal.pone.0086320 (PMC3897677; doi:10.1371/journal.pone.0086320)
Supplement: Figure S1 — Repeated TGA/DSC scans of human insulin amyloid samples. (PDF) [file pone.0086320.s001.pdf]

On the heat stability of amyloid-based biological activity: insights from thermal degradation of insulin fibrils.

(Thermal degradation of insulin fibrils)

Weronika Surmacz-Chwedoruk, Iwona Malka, Łukasz Bożycki, Hanna Nieznańska, Wojciech Dzwolak

Supporting Information

Repeated TGA/DSC scans of human insulin amyloid samples: demonstration of the high degree of reproducibility of the data in Figure 2.

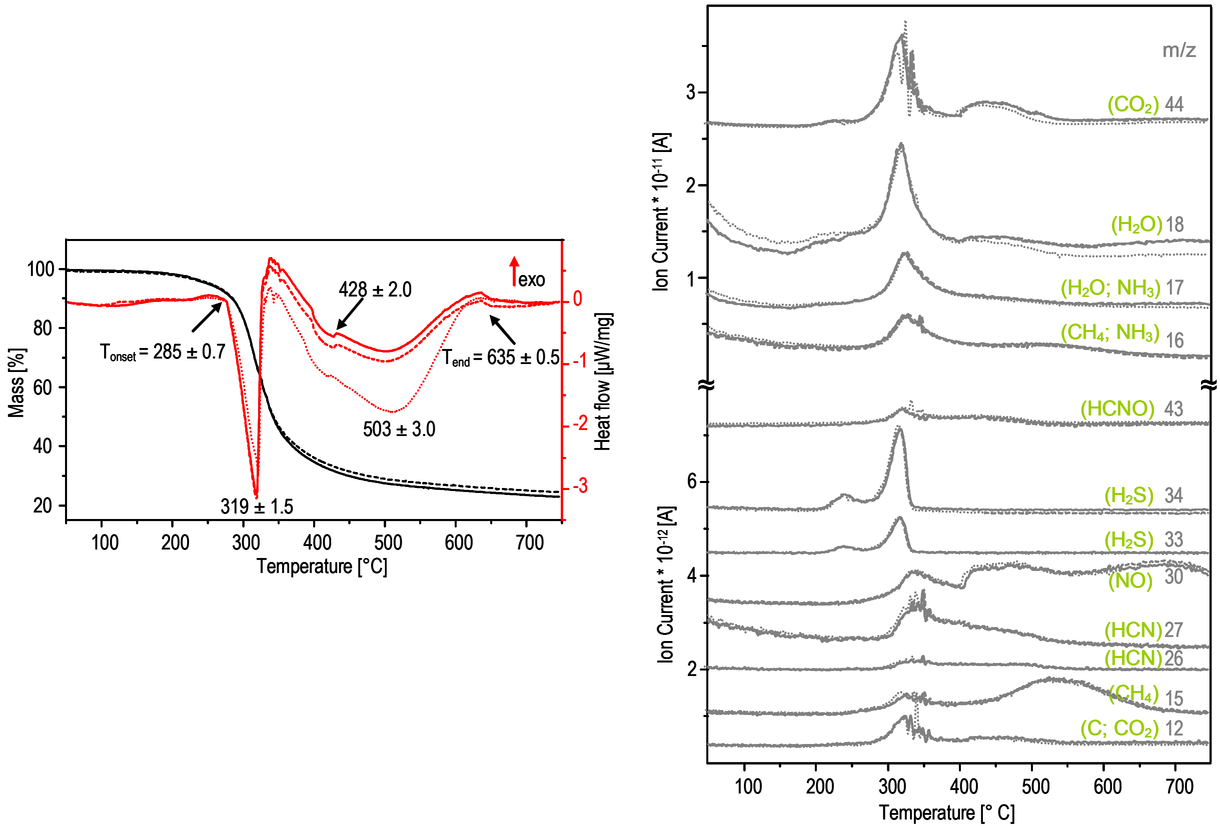

Figure S1.

Figure S1 shows several overlapping TGA and DSC traces (left panel: black and red lines, respectively) and corresponding MS profiles of released gases (right panel) for repeated experiments depicted in Figure 2 of the main article.
